# Metronidazole-Induced Encephalopathy in a 16-Year-Old Girl with Crohn’s Disease: Case Report and Review of the Pediatric Literature

**DOI:** 10.3390/children9091408

**Published:** 2022-09-17

**Authors:** Karolina Rybak, Aleksandra Warchoł, Łukasz Drobczyński, Aleksandra Banaszkiewicz

**Affiliations:** 1 Department of Pediatric Gastroenterology and Nutrition, Medical University of Warsaw, 02-091 Warsaw, Poland; 2 Department of Pediatric Radiology, Medical University of Warsaw, 02-091 Warsaw, Poland

**Keywords:** antibiotic treatment complication, cerebellar syndrome, gait disturbance, inflammatory bowel disease, magnetic resonance imaging, neurologic complication, speech disorders

## Abstract

Metronidazole-induced encephalopathy (MIE) is a rare and unpredictable complication that is most commonly reported in adults. Here, we present the case of MIE in a patient treated with rectal, oral, and intravenous metronidazole. This is the first case of MIE reported after suppositories. A 16-year-old girl with Crohn’s disease treated with mesalazine and exclusive enteral nutrition was operated on due to perianal fistulas and abscesses. She received oral metronidazole for 25 days and rectal metronidazole for 15 days as an adjuvant before surgery. Moreover, 2.5 g of intravenous metronidazole was administrated during the perioperative period. The second day after the surgery, symptoms of cerebellar syndrome appeared. She presented with an inability to coordinate balance and gait. Although she showed accurate verbal responses, her speech was slow, slurred, and scanning. The finger–nose test was positive. The T2-weighted magnetic resonance imaging revealed an increased symmetrical signal within the dentate nuclei of the cerebellum and in the corpus callosum. The changes were characterized by restricted diffusion. Based on the clinical picture and magnetic resonance imaging findings, MIE was diagnosed. Treatment with metronidazole was discontinued. The cumulative dose of metronidazole that she received for 29 days was 54 g: 38 g p.o., 13.5 g p.r., and 2.5 g i,v. The first symptoms appeared on the 28th day of antibiotic therapy after receiving 52 g of metronidazole. The neurological symptoms resolved after six days; however, three days after the resolution, paresthesia appeared in the distal phalanges of both feet and lasted for approximately two months. Our report highlights that neurologic symptoms related to metronidazole treatment should raise the suspicion of MIE.

## 1. Introduction

Metronidazole-induced encephalopathy (MIE) is a rare and unpredictable complication that is reported most frequently in adults. The most common symptoms in children are gait instability, altered mental status, seizures, visual disturbances, limb dyscoordination, dizziness, and vomiting [1,2,3,4,5,6,7,8,9,10,11,12,13]. Here, we present the case of MIE in a patient with Crohn’s disease (CD) treated with rectal, oral, and intravenous metronidazole. This is the first report of MIE after the use of suppositories.

CD is a chronic disorder that may affect all gastrointestinal tract segments. Strictures, fistulas, and abscesses are complications of an ongoing inflammatory process and lead to bowel damage and disability. The goal of treatment is to achieve symptomatic response and remission, normalization of serum and fecal biomarkers, endoscopic healing, normalized quality of life, and absence of disability [14,15].

## 2. Case Presentation

Here, we present a case of a 16-year-old girl with CD that manifested with diarrhea and abdominal pain and was treated with exclusive enteral nutrition and mesalazine. One month after diagnosis, she reported pain in the perianal area and purulent discharge. Therefore, a transrectal ultrasound and pelvic magnetic resonance imaging (MRI) was performed. Imaging tests showed a complex fistula with branches passing through the intersphincteric space, multiple abscesses, and inflammation in the distal part of the rectum. According to the Paris Classification, the disease phenotype was changed from the initial A1bL3L4aB1G1 to A1bL3L4aB3G1. 

Due to an extensive purulent process, metronidazole in oral and rectal form was used as an adjuvant before surgery and anti-TNF treatment. The patient received 500 mg of oral metronidazole three times a day for 25 days and 500 mg of rectal metronidazole two times a day for 15 days until surgery as well as 500 mg of intravenous metronidazole on the day of surgery. The surgery proceeded without complications. The next day, the girl did not present worrying symptoms; 500 mg of oral metronidazole was administered three times for that day. 

The second day after surgery, the patient’s general condition deteriorated. The girl had swallowing problems, and thus intravenous metronidazole was administrated again. She presented with an inability to coordinate balance and an unstable gait. Though she showed accurate verbal responses, her speech was slow, slurred, and scanning. The finger–nose test was positive. She showed muscle weakness in all limbs. No other abnormalities in the physical examination were found. In the laboratory tests, the CRP was slightly increased (1.48 mg/dL). 

**Figure 1:**
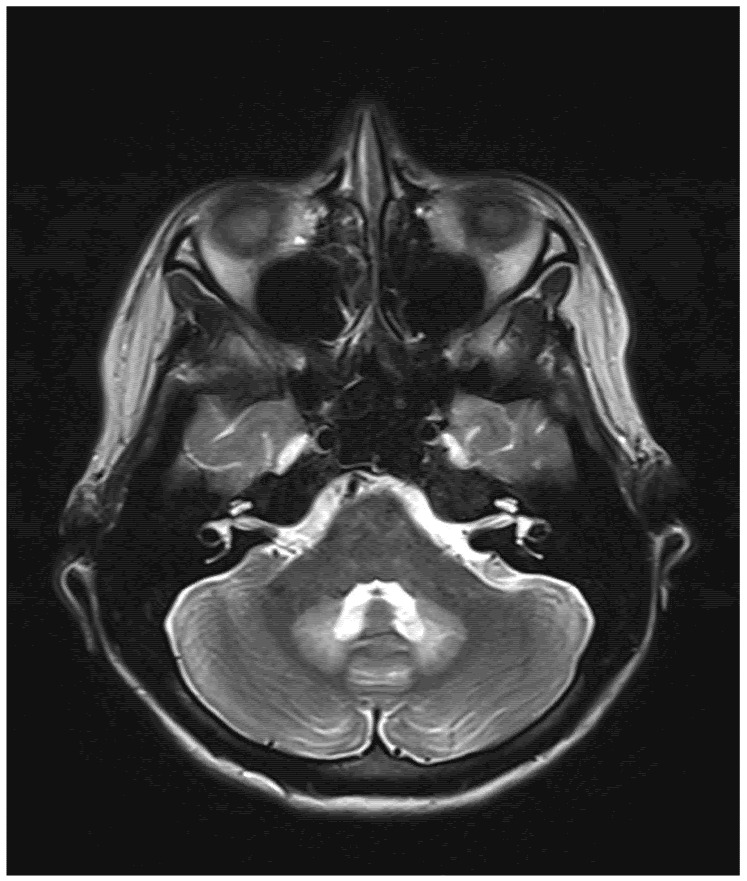
T2-weighted MRI axial view showing signal hyperintensity within dentate nuclei.

**Figure 2:**
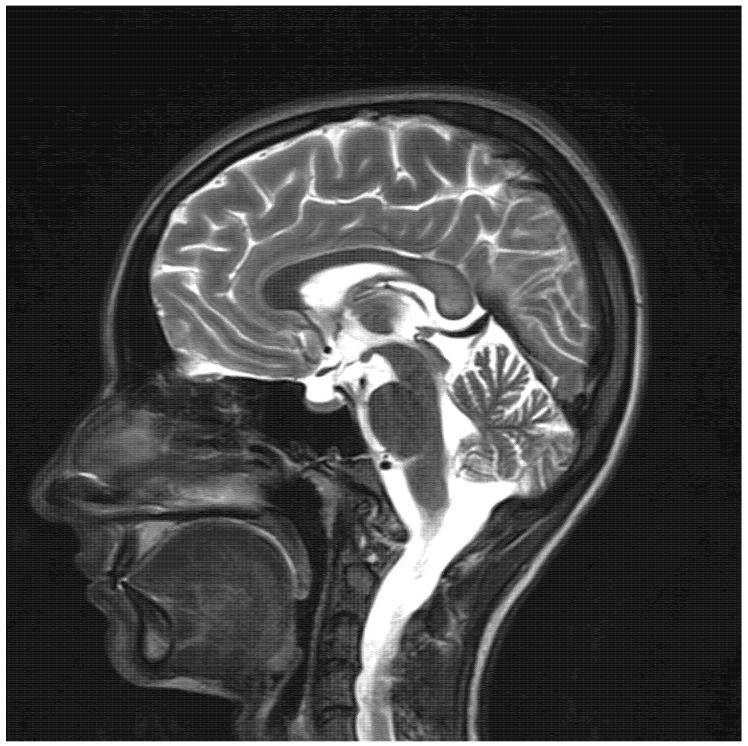
T2-weighted MRI sagittal view showing signal hyperintensity within the splenium of the corpus callosum.

**Figure 3:**
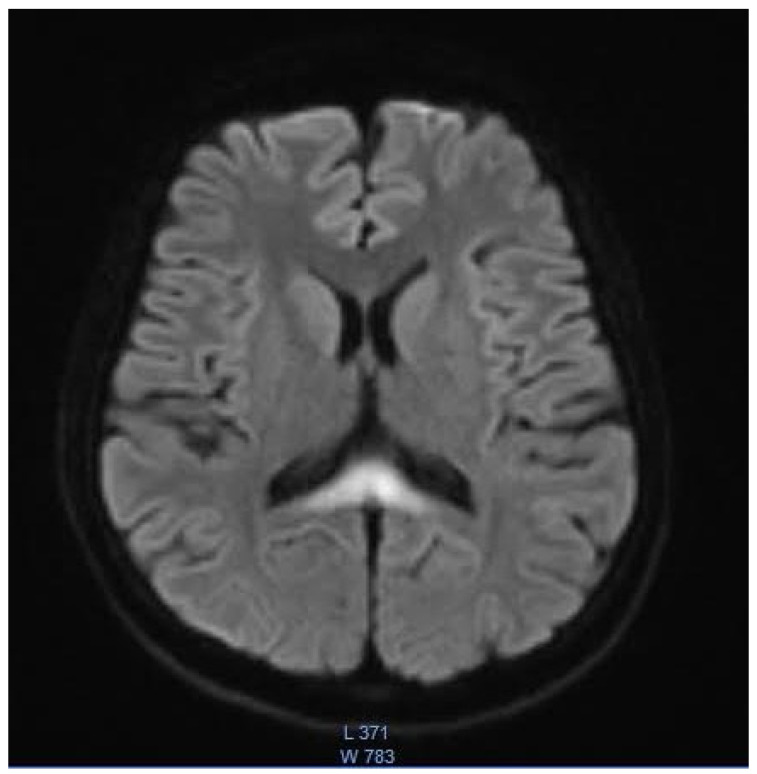
The DWI reveals a high-signal intensity within the splenium of the corpus callosum.

**Figure 4:**
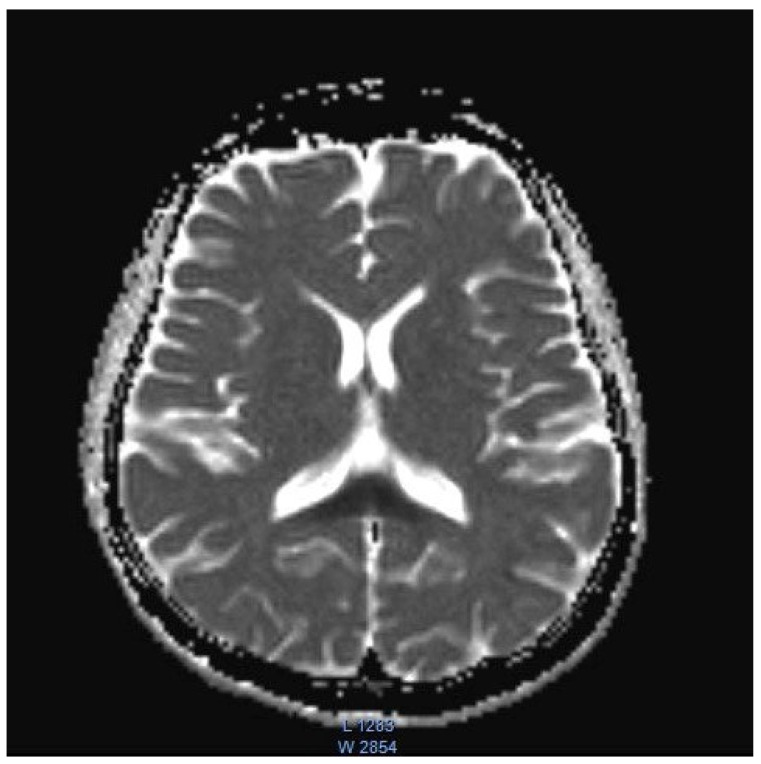
The reduced ADC values within the splenium of the corpus callosum.

The remaining laboratory tests were within the normal range, including the liver enzymes and sodium and potassium serum levels. The EEG test results were normal. As there were no abnormalities in computed tomography, an MRI was performed. The T2/TIRM images showed an increased symmetrical signal in the corpus callosum and the cerebellum’s white matter and dentate nuclei. (Figure 1 and Figure 2). The changes in the splenium of the corpus callosum were characterized by high-signal intensity on diffusion-weighted imaging (DWI) (Figure 3) with corresponding reduced apparent diffusion coefficient (ADC) values (Figure 4).

Based on the clinical picture and MRI findings, MIE was diagnosed. Treatment with metronidazole was discontinued. The cumulative dose of metronidazole that she received for 29 days was 54 g: 38 g p.o., 13.5 g p.r., and 2.5 g i.v. The first symptoms appeared on the 28th day of antibiotic therapy, after receiving 52 g of metronidazole. Neurological symptoms resolved six days after cessation; however, three days after the resolution, paresthesia appeared in the distal phalanges of both feet and lasted for approximately two months.

## 3. Discussion

Metronidazole is a 5-nitroimidazole antibiotic with activity against anaerobic bacteria and protozoa. In patients with CD, metronidazole is recommended in treating perianal fistulas, managing abdominal abscesses, and in the postoperative period [14]. Most cases of MIE are reported in adults. To our knowledge, 13 cases in children have been published thus far, of which three children had CD [1,2,3,4,5,6,7,8,9,10,11,12,13]. They all had resolution of symptoms or markedly improved with the discontinuation of metronidazole. We included the essential information on these cases in Table 1.

MRI usually shows hypertensive lesions in the dentate nuclei, midbrain, corpus callosum, and medulla oblongata. Lesions are nearly always bilateral and symmetric, without pathologic enhancement [12]. In previous pediatric cases, MRI showed complete or partial resolution of lesions.

The pathomechanism of MIE is still not fully understood; the proposed hypotheses include disruption of the energy–metabolic pathways, loss of GABAergic inhibition, and vasogenic and cytotoxic edema [16].

Our patient received a high dose of 54 g of metronidazole, including 38 g p.o., 13.5 g p.r., and 2.5 g i.v. However, there appears to be no relationship between the MIE, metronidazole single dose, and the cumulative dose [16,17]. MIE symptoms may occur after receiving a small amount (even 250 mg) [18] or after many years of use of metronidazole [19]. Only three published pediatric cases provided information about the cumulative dose, which amounted to 4, 12, and 1378.8 g [1,8,9]. Among adult patients, the cumulative doses of metronidazole ranged from 5 to 2000 g with an average of 125.7 g [16].

This is the first case of MIE in a patient treated with rectal metronidazole. The suppositories were combined with an oral form. The patient received only intravenous metronidazole on the day of surgery and on the second and third days after surgery. The absorption and overall exposure of rectally administered metronidazole is comparable to when administered orally [20]. Even gel with metronidazole applied to pressure ulcers can cause encephalopathy [21]. Thus, the suppositories may have contributed to our patient’s neurological condition.

Cerebellar and brainstem deficits occurring close to metronidazole treatment should raise suspicions of MIE. However, several other diseases should be included in the differential diagnosis. A deficiency of thiamine causes Wernicke’s encephalopathy. Clinical findings include a wide range of symptoms; however, the most characteristic are ataxia, gait instability, altered mental status, and ocular abnormalities [22]. 

Although Wernicke’s encephalopathy is most often caused by alcohol abuse, non-alcoholic cases of patients with CD and malabsorption have also been reported [23,24]. Similarly, Marchiafava–Bignami disease is associated with alcoholism and/or malnutrition and thiamine deficiency. The condition is manifested by apathy, depression, and aggressive behavior with interhemispheric disconnection syndrome’ related to apraxia, ataxia, visual dyslexia, loss of consciousness, seizures, mental confusion, and psychosis [25]. Wernicke’s encephalopathy and Marchiafava–Bignami disease usually differs from MIE in MRI.

Other types of encephalopathy, such as hepatic or antibiotic-associated, should also be considered. The dominating symptoms of encephalopathies induced by penicillin and cephalosporins are myoclonus and seizures. In these, the MRI is normal; however, the EEG shows abnormalities. Encephalopathies caused by procaine penicillin, sulfonamides, fluoroquinolones, and macrolides usually manifest in psychosis with a normal MRI [26].

Central nervous system infection, cerebral venous thrombosis, acute disseminated encephalomyelitis type of demyelination, posterior reversible encephalopathy syndrome, and non-convulsive epileptic status may manifest as encephalopathy in patients with IBD. They are also at risk of developing neurological complications because of the medications [27].

Canavan disease, maple syrup urine disease, glutaric aciduria type 1, and Leigh syndrome are rare, congenital diseases that can cause T2/FLAIR-hyperintense lesions in the dentate nuclei [28]. Despite some common symptoms, the clinical picture usually differs from MIE [29,30,31]. Laboratory and genetic tests are critical in differential diagnosis [32,33,34].

There is no specific treatment for MIE. Usually, patients recover spontaneously within days of stopping drug administration; therefore, treatment is primarily symptomatic [16]. Plasmapheresis was successfully used in one child with cortical visual impairment [4]. A 57-year-old patient with MIE that worsened after drug cessation was successfully treated with high-dose intravenous methylprednisolone [35]. However, in a 17-year-old boy, a 5-day course of high-dose intravenous methylprednisolone was not effective until the cessation of metronidazole [13].

## 4. Conclusions

In conclusion, we reported a case of a patient with CD who presented symptoms of encephalopathy during treatment with metronidazole. As metronidazole is a widely used antibiotic, neurologic symptoms, especially cerebellar deficits, occurring closely to metronidazole treatment should raise suspicions of MIE.

## Figures and Tables

**Table 1:** Case reports of MIE in children.

Author, Year, Country	Age (Years), Sex	Indication	Cumulative Dose of Metronidazole	Days to First Symptoms; Total Duration of Treatment	Symptoms and Clinical Findings	MRI Findings	Follow Up MRI	Outcome
Bailes, 1983, USA [1]	12, M	Perforated appendicitis	4 g	4; 4	Altered mental status; seizures	N/A	N/A	Resolution
Bates, 2015, USA [2]	36-day-old, M	Mother’s vaginosis (utero exposure)	N/A	N/A; N/A	Hypothermia; bradycardia; failure to thrive; decreased tone and strength	T2: symmetrical hyperintensity in the dentate nuclei	Not done	Resolution
Cecil, 2002, USA [3]	17, M	Crohn’s disease	N/A	N/A; N/A	Gait instability; Polyneuropathy; visual disturbance; tremor	T2: Symmetrical hyperintensity in the substantia nigra, red nucleus, globus pallidus, the putamen, caudate body, caudate heads and medial thalami	Near complete resolution	Near complete resolution
Chang, 2021, USA [4]	14, M	Clostridium difficile enterocolitis	N/A	N/A; N/A	Altered mental status; stiffening of 4 extremities; visual disturbance; slurred speech; gait instability	T2: symmetrical hyperintensity with corresponding diffusion restriction on DWI in posterior frontal, parietal, and occipital periventricular white matter and splenium of the corpus callosum	Near complete resolution	Near complete resolution
Chatzkel, 2010, USA [5]	15, F	Crohn’s disease	N/A	7; N/A	Ataxia; dysmetria	T2: Symmetrical hyperintensity in the dentate nuclei	Resolution	N/A
Gaye, USA, 2007 [6]	Teenager, M	appendectomy	N/A	N/A; N/A	Unresponsiveness; respiratory distress; decerebrate posturing	Left parietal flair signal	N/A	Resolution
Kafadar, 2013, Turkey [7]	3, M	amoebiasis diarrhea	N/A	14; N/A	Loss of vision; ataxia, dizziness	Normal	N/A	Resolution
Omrani, Iran, 2020 [8]	11, M	Febrile bloody diarrhea	12 g	N/A; N/A	Tinnitus; hearing loss; aggressive behavior; generalized dystonia; generalized tonic-clonic seizure; decreased level of consciousness.	T2: Symmetrical hyperintensity in dentate nuclei, substantia nigra, globus pallidi, splenium of the corpus callosum, and centrum semiovale	Improvement	Partial improvement
Patel, USA, 2020 [9]	8, M	Prophylaxis after small bowel transplantation	1378.8 g	Three years; three years	Ataxia	T2: Symmetrical hyperintensity in the dentate nuclei, inferior olivary nuclei, putamen, and corpus callosum	Resolution	Resolution
Starrs, 2021, USA [10]	12, M	Clostridium deficile infection	N/A	75; 75	Vertigo; nausea; vomiting; ataxia; gait instability	T2: Symmetrical hyperintensity in the dentate nuclei	N/A	Resolution
Sudan, 2016, India [11]	14, M	Acute abdominal pain	N/A	3; 5	Dysarthria; altered mental status; seizures.	T2: symmetrical hyperintensity in the optic tracts, dorsal midbrain, inferior olivary nuclei, peri-aqueductal white matter, superior and inferior colliculi, superior cerebellar peduncle, dentate nuclei, medulla oblongata, and cervical spinal cord segment extending from the cervicomedullary junction to C6-C7 level DWI: restricted diffusion in the splenium of the corpus callosum	N/A	Resolution
Sun, 2019, USA [12]	11, M	Fusobacterium menigitis	N/A	3 months; N/A	Vomiting; dizziness; vertigo; gait instability; bilateral lower extremity paresthesia	T2: Symmetrical hyperintensity in the dentate nuclei, dorsal pons, and medulla. DWI: no restricted diffusion	Resolution	Resolution
Yazdani, 2019, USA [13]	17, M	Chronic diarrhea	N/A	N/A	Gait instability; abnormal unilateral lean	T2: Symmetrical signal hyperintensity in dorsal pons, dentate nuclei, dorsal medulla	Improvement	Resolution

F—Female; M—Male; N/A—Information not available in the cited paper; T2—T2 weighted MRI; and DWI—Diffusion-weighted imaging.

## Data Availability

Not applicable.
